# Assessment of the quality of interdisciplinary communication (CritCom): evaluation and refinement of a center summary report

**DOI:** 10.3389/fonc.2024.1384597

**Published:** 2024-06-11

**Authors:** Lara Counts, Jocelyn Rivera, Parima Wiphatphumiprates, Maria Puerto-Torres, Kim Prewitt, Douglas A. Luke, Dylan E. Graetz, Sara Malone, Asya Agulnik

**Affiliations:** ^1^ Brown School, Washington University in St. Louis, St. Louis, MO, United States; ^2^ Department of Pediatrics, Hospital Infantil Teletón de Oncología (HITO), Querétaro, Mexico; ^3^ Rhodes College, Memphis, TN, United States; ^4^ Global Pediatric Medicine, Critical Care, St. Jude Children’s Research Hospital, Memphis, TN, United States

**Keywords:** report, interdisciplinary communication, deterioration, pediatric oncology, critical care

## Abstract

Communication failures among clinicians in the ICU (intensive care unit) often lead to worse patient outcomes. CritCom is a bilingual (English and Spanish) tool to evaluate the quality of interdisciplinary communication around patient deterioration for pediatric oncology patients. The use of reports, such as the CritCom report, as dissemination methods lead to quicker knowledge translation and implementation of research findings into policy. Nurses and physicians at participating centers who care for patients at risk of deterioration completed the CritCom survey and center-specific reports were generated to communicate CritCom results. Focus groups were conducted with clinicians receiving CritCom reports in both English and Spanish to evaluate report clarity and usability. Participants found the reports to be useful and described the writing and design as clear and specific. Participants provided feedback to improve report design and requested actionable steps to improve communication at their center. Feedback illustrated that the report was easy to interpret and a useful way to disseminate information. Participants noted the utility of the report, illustrating that the use of reports can be a useful method to disseminate research findings back to participants in a way that is applicable to the local context. Communicating research findings through reports can minimize the significant time lag in knowledge translation and provide participants with actionable steps to implement in their setting.

## 1 Introduction

Outcomes for critically ill patients improve when clinicians work together and communicate effectively as a team (1–3). Communication between clinicians is particularly important in the care of children with cancer, who are at higher risk of deterioration and subsequent mortality (2, 3). High-quality interdisciplinary communication has been linked to earlier recognition of adverse events and decreased mortality (4). Communication failures, however, often impact clinicians understanding of patient care plans (4), resulting in worse patient outcomes by delaying treatment and causing injury (5). Thus, enhancing interprofessional communication is important to improve patient outcomes and quality of care delivery (4, 6).

Barriers to teamwork and communication between clinicians include feeling disempowered to speak up, issues with hierarchy, and negative interpersonal communication (7). Few studies have addressed the quality of team communication, especially in resource-diverse settings. The CritCom tool, developed to fill this gap, is a new reliable and valid bilingual survey to assess the quality of interdisciplinary communication around patient deterioration for pediatric oncology patients (8). CritCom is an anonymous electronic provider survey that evaluates communication between clinicians across six domains: actionable, clarity, tone, collaboration and teamwork, leadership, and empowerment. CritCom was initially piloted at 42 hospitals in 22 countries among clinicians who care for children with cancer at risk of deterioration (9). For centers with three or more participants, a center-specific report was created to summarize responses and communicate results. This study explains the development of the report, which includes the initial drafting, review using focus groups, and revision of the report. This study also evaluated the clarity and usability of the CritCom center reports.

Recently, emphasis has been placed on creating dissemination efforts that are adaptable to local contexts, engaging stakeholders and encouraging continuing collaboration between researchers and participants (10). The use of reports can aid in quicker knowledge translation, closing the significant gap between research and practice (11). Further, reports must be developed in a clear manner, tailored to the stakeholder with clear and actionable messages (12). If research findings are not disseminated, then the research efforts themselves are largely considered a waste of effort and resources (13).

Timely report development, publication, and dissemination are important to quickly inform survey participants and hospital administration of the strengths and weaknesses of communication at their center, which can ultimately be used to implement policy focused on interprofessional communication in critical care settings, improving patient outcomes.

## 2 Methods

### 2.1 Report development

**Figure 1:**
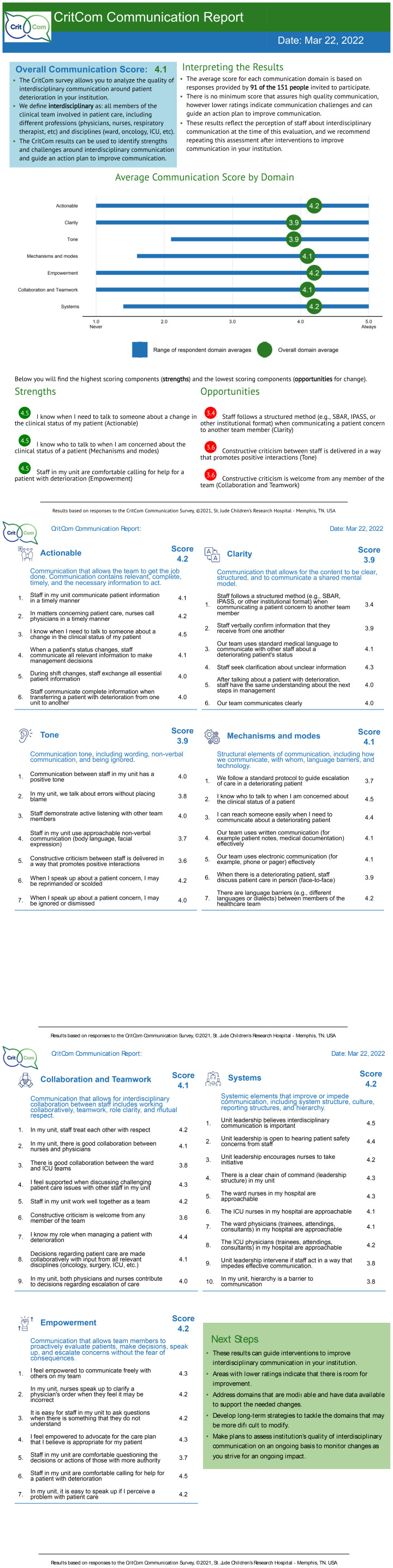
CRITCOM report.

For centers with three or more participants, a report summarizing all staff responses was generated (see **Figure 1**) in English or Spanish based on prior experience with center-level reports of staff assessments (14). The report described performance in each domain (average and range), the overall communication score (average of all domains), list of strengths and opportunities (highest and lowest scoring items), detailed performance in each survey item, and suggestions for next steps. This report was modeled after another report created and used by this team for a prior study (15). A first draft of the CritCom report was drafted by the study team and reviewed by all study team members. The CritCom report was distributed to all participants at each center.

### 2.2 Report assessment

The CritCom report was assessed via focus groups consisting of participants from various centers. Participants were recruited among all individuals who completed the survey and received a hospital-based report. Focus groups were organized by participant profession (nurse vs. physician) and language (English vs. Spanish), with a total of four focus groups. The focus groups were structured using a facilitator guide to evaluate participant understanding about their center-specific report, as well as communication in their hospitals (see **Additional File 1**). The guide was initially developed in English based on prior work (14). A pilot focus group was conducted with five participants from St. Jude representative of the target audience. The guide was then revised based on feedback and translated to Spanish by bilingual team members (JR and MPT).

**Figure 2:**
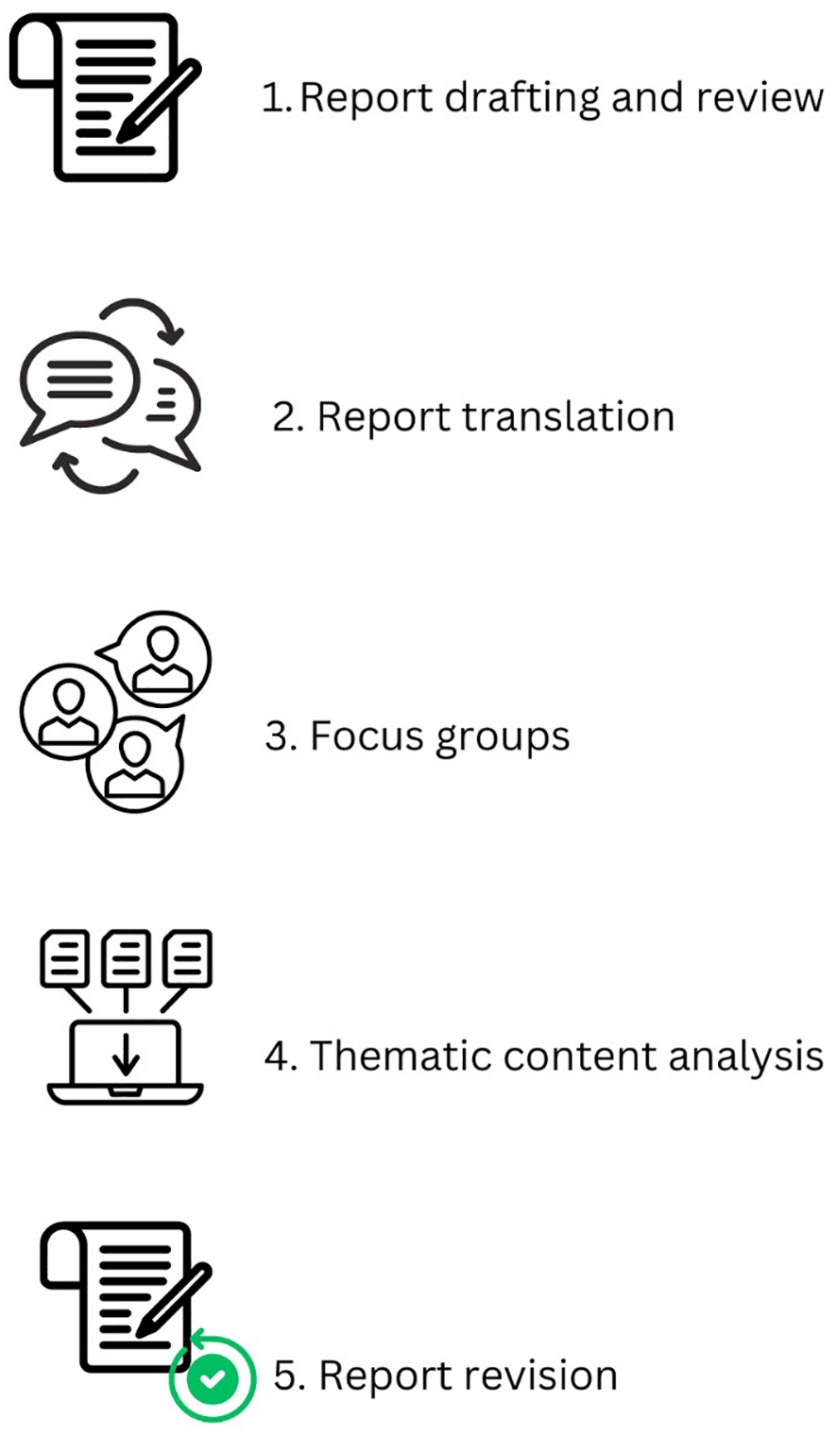
Report development and assessment process.

Focus groups were held via the web-conferencing platform Zoom. Participants were encouraged to participate with their video and engage as during an in-person discussion. Two individuals (PW and LC) who were not involved in CritCom report development facilitated the English focus groups. Bilingual members of the team (JR and MPT) facilitated the Spanish focus groups. Focus groups were audio-recorded, then were translated and transcribed by a professional service. Transcripts were deidentified and uploaded into MAXQDA for thematic analysis (16, maxqda.com). A codebook was developed from previous work and iteratively revised through review of two transcripts (see **Additional File 1**) (17). Two investigators (PW and LC) coded all transcripts with discrepancies resolved by two adjudicators (AA and SM). Thematic content analysis focused on participant experiences with communication and CritCom report feedback. The development and assessment process is depicted in **Figure 2**.

## 3 Results

Focus groups consisted of 11 English-speaking participants from five countries and 12 Spanish-speaking participants from four countries; these were 57% physicians and 43% nurses with a primary work area in the ward (65%) and intensive care unit (ICU) (35%). Identified themes included experiences with communication in their setting, report interpretation, and recommendations for improvement (**Table 1**).

**Table 1:** Focus group feedback.

Focus group feedback		
Theme	Code	Example quote
Experiences with communication in their setting	Communication challenges	“I also think that sometimes the other person to whom we are sending that message, does not receive it with the same importance in the way that we are taking the deterioration of the patient and maybe on those occasions is when the patient and the communication is lost.”
	Good communication	“But most of all I feel that it is the trust and the empowerment that one has on the tool to communicate with the doctor.”
	Impacts on patient care	“That is where the patient could be a transfer from intensive care when we could have performed actions before in the service. I think that bad communication between all would affect a lot.”
	Other communication	“And the focus area, because the communication between health workers, physician, nurses, between unit and the unit is very important.” “We know when to talk when need to transfer patients from one place to another there’s an adequate amount of concern over the critical state of the patient and all of those mechanisms are functioning well, effective communication.”
Report interpretation	Ease of interpretation	“I think that the graphs are very clear and that the extent of the report is obviously enough.”
	Report or score interpretation	“I would interpret that in general we are not doing too bad but we can improve in many of the aspects and go through the pages to see what we have to work on to improve.”
	Report use	“I consider that the report is good. It gives us the three … well it gives us the opportunities and strengths and under opportunities, we can guide ourselves or we can support ourselves from there to make improvement projects and later if we do the survey again to be able to measure how much we did, what we worked on and in what other things we can continue working.”
	Seeking additional guidance	“So, I’m not sure that we’ll be able to take specific action without a little bit more guidance based on these areas with room for improvement.”
	Written material	“I think that it’s very well written and summarized in a form that is very quick and easy because it provides us with all the information that has been collected in the survey so I think that it’s okay and just as [Doctor 3] said the extent of it is correct.”
	Domain graph	“I think that the graphs are very clear and that the extent of the report is obviously enough”
	Strengths and opportunities	“I think particularly the part that highlights the strengths and opportunities with the highest and lowest scores was helpful in just pulling that information to the front, but then having the opportunity to read and go deeper into the specific questions for each domain was up with scores was also helpful.” “Yes, I agree. It is very well broken down in that each item that we are evaluating or that they evaluated us and where it tells us where we had the highest or lowest score of each area … let’s say … team collaboration is where we are not doing well. We get to see each item where we were evaluated, and each question is very well explained, and it gives us as the idea of what we have to work. For me, it is quite good.”
	Second and third pages	“It’s not only about the opportunities that are highlighted two and three but also that we can break down every single item and see what are the aspects that we can also improve on.”
	Other components	“I believe, apart from everything that is being said, that in regards to the images that appear at each of the subtitles, I don’t know, maybe they should be a little larger or in color.”
	Overall report	“To me I do not think that anything else is needed. To me, it was complete and digestible.”
Recommendations for improvement	Confusion with the survey details	“Is it going to be for permanent staff or only for rotating substitute personnel? I think it would also be like specifying who the survey is addressed to.”
	Report feedback	“Yeah, I think just looking at the overall communication score, you can’t really tell much from it.” “What are some interventions or even further assessments that can be done to look at those areas of opportunities? So what can you give us to help improve the way the constructive criticism is received?”

Participants noted multiple examples of poor communication in their clinical settings: “*Maybe with nurse and physicians is better, but don’t think the multidisciplinary team we don’t have the same language for everyone.”* They also reported instances of good communication: “*how we communicate with other people not only because it is a doctor, a nurse, or staff member. We have to go all on the side of good communication for us to have the results … well positive to be able to assist the patient*.” Poor and good communication were noted to impact patient care: “*Well, I consider that if we take a long time to report on the patient’s status, we also lengthen the patient’s treatment time.”*

Overall, participants found the CritCom report to be clear, specific, and helpful to the strengths and weaknesses of communication between clinicians at their center. “*It’s very well designed and the information is adequate, concrete and at the same time extensive within two to three pages with a lot of graphics.*” Participants described the report scores and graphs easy to interpret. “*We just have to improve in the few points that we failed and not drop points in those where we did well.*” Participants also recognized the utility of the report to develop strategies to enhance communication within and between units: “*What we must work on and where we can continue to apply the knowledge that we already have but only reinforce them and have greater empowerment which is where we scored the lowest.*”

Additionally, participants offered several recommendations to improve the report, such as providing more information about participant demographics. Many also noted a need to include actionable steps: “*It would also be good to add a box with recommendations from their experience that they elaborated this survey to improve that communication in at least the areas where the scores were the lowest*.”

## 4 Discussion

This study describes the evaluation of the CritCom report to promote understanding of study findings by centers participating in the CritCom assessment. Our findings demonstrate that participants found the report to be clear, usable, and useful to visualize and understand their results.

The time lag between research and implementing findings into practice is too long (10, 18). Further, the percentage of research results that are implemented into practice is low, at approximately 14% (13, 18). Dissemination of information is necessary to adopt research findings into clinical practice (13).

Adapting research findings through the creation of reports is helpful to minimize this time lag by quickly transferring information from the researchers back to the clinical setting. Clear, easy to read, and descriptive reports describing communication can help hospital administration and unit leaders pinpoint areas of strength and weakness that can be targeted for intervention. Feedback from this report illustrates that participants could use the report to take actionable steps to improve communication at their hospital.

Studies have found that simply publishing research findings is often ineffective in actually changing practice, and thus the gap remains between research and practice (11). Targeted dissemination efforts, such as the CritCom report, are useful methods of translating information back into the hands of clinicians.

This work represents an example of how research findings can be made available to participants to promote local quality improvement and actionable change. Clinicians, researchers, and administrators can utilize the CritCom report to interpret CritCom results and improve interdisciplinary communication and, subsequently, patient outcomes. For example, the report can inform center-specific trainings or other strategies to improve the areas of communication that scored low. Providing clear and contextually appropriate reports of research findings allows participants to use study results to advocate to their hospital administration for local change.

This study has several limitations. Only nurses and physicians were invited to participate; members of the interprofessional team (respiratory therapists, etc.) were not included, as these roles did not exist at all centers. Additionally, this study was limited to English- and Spanish-speaking participants. This limits the generalizability of our findings regarding CritCom report usability to other professions and languages; future work should include these groups.

In summary, participant feedback illustrates that the CritCom report successfully provided clear and relevant findings regarding communication quality at each center. Using dissemination methods such as a summary report is useful to provide participants timely and actionable research data to inform strategies to improve team communication in their setting.

## Data availability statement

The raw data supporting the conclusions of this article will be made available by the authors, without undue reservation.

## Ethics statement

Ethical review and approval were waived for this study due to determination of non-human subjects research as defined by the Common Rule at 45 CFR 46.102(I) and the Office for Human Research Protections (OHRP). It is under an exempt IRB from St. Jude Children’s Research Hospital. The written informed consent was waived by IRB from St. Jude Children’s Research Hospital.

## Author contributions

LC: Writing – review & editing, Writing – original draft, Formal analysis, Data curation. JR: Writing – review & editing, Methodology, Funding acquisition, Data curation, Conceptualization. PW: Writing – review & editing, Formal analysis, Data curation. MP: Writing – review & editing, Project administration, Formal analysis, Data curation. KP: Writing – review & editing, Project administration, Conceptualization. DL: Writing – review & editing, Funding acquisition, Conceptualization. DG: Writing – review & editing, Methodology, Conceptualization. SM: Writing – review & editing, Supervision, Methodology, Funding acquisition, Conceptualization. AA: Writing – review & editing, Resources, Methodology, Investigation, Funding acquisition, Conceptualization.
