# Supplementary material for: Assessment of the quality of interdisciplinary communication (CritCom): evaluation and refinement of a center summary report
Source: Front Oncol. 2024 Jun 11;14:1384597. doi: 10.3389/fonc.2024.1384597 (PMC11234842; doi:10.3389/fonc.2024.1384597)
Supplement: Supplementary file 1 [file Table_1.docx]

**Assessment of the quality of interdisciplinary communication (CritCom): development and refinement of a center summary report**

**Table of Contents:**

| **Content** | **Page** |
| --- | --- |
| Table S1: Facilitator guide (English) | 2 |
| Table S2: Codebook | 6 |

**Table S1: Facilitator guide (English)**

| **Welcome** |
| --- |
| Welcome to this focus group; I would like to thank you for taking the time to talk with us today. The objective is to discuss the report based on the Quality of Interdisciplinary Communication (CRITCOM) survey between providers, which you completed.  My name is *<state your name, role, and institution>,* and I will be the facilitator for our conversation today.  ***[if a co-facilitator is present]***  We're joined today by *<Insert Name, role, and institution>,* who will serve as a co-facilitator, take notes, and assist us in ensuring everything runs smoothly from a technical standpoint. *<Co-facilitator>* will be aware of your comments in the chat to address any technical problem (audio, difficulties seeing the video, etc.). Remember to keep your camera on as much as possible. |
| **Description** |
| We are conducting a series of focus groups with clinicians who completed the Quality of Interdisciplinary Communication (CRITCOM) to discuss their results report.  Our goal today is to ensure that everyone has the opportunity to share their comments and feedback to evaluate and improve this report. (Might be useful to show the report for a moment, to guide them) |
| **Rules to follow** |
| Before we begin, I would like to review some basic rules for this focus group:   - Make sure you have the CritCom Report that was provided to you on hand. if you have not received your report, please let us know and we will make sure someone from our team send it after our session ends - This session will be recorded, and the audio will be transcribed and de-identified for analysis - For this session, we will identify and refer to each participant using their first name to avoid hierarchies and facilitate communication. - There are no right or wrong answers to the questions we will ask today. We want to hear about your ideas, experiences, and opinions, feel free to share your thoughts openly. - You do not have to wait to be called to participate in the round of questions. It is an open discussion, so that you can comment anytime. |
| **Technical considerations to consider** |
| - - Please keep your camera on throughout the session.   - Remember to keep your microphone muted until you are ready to speak; activate your microphone whenever you want to comment or say something.   - Please use the "chat" function only to communicate technical problems as we want you to express your comments verbally to the group. |
| **Doubts before proceeding** |
| - Do you have any questions regarding the rules or technical matters before we start? |
| **Presentation of the participants** |
| As I mentioned, my name is <facilitator name>. I will have each of you briefly introduce yourself to the group. In this section, I will call you so that each of you can say your name, place of origin, clinical role, and how many months or years of experience you have providing medical care.  *(The facilitator will lead this part of the session with the help of the list of participants).* |
| **Introduction to the round of questions:** |
| For the remainder of this session, please remember that you do not have to wait to be called on to speak. Please, express your opinion or comment when you consider it appropriate. As we get started, I wanted to remind you all that this project focuses on the measurement of communication between units and clinicians within the hospital. For example, this could involve nurse and physician communication across both the oncology ward and the ICU.  What is the easiest or most difficult aspect of communication in your unit?  How do you feel like your communication with other clinicians impacts the care that is provided to patients? |
| **Overall Appearance:**  ***(Request that the co-facilitator shares their screen with the image of the report)*** |
| We're also interested in your opinion on the best way to present the information in the report so that it is easy to read and understand..  As you know, the report is divided into the following sections:  - Score  - Written text  - Domain graphics  - Strengths and Opportunities, and  - Details on the second and third page   1. Is the information organized in a way that makes sense?    1. What would you improve?    2. Is there something that it is missing from this report? 2. Is there anything about how the report looks that you find confusing?    - Score review box?    - Written text?    - The domain averages graph?    - Strengths and Opportunities?    - Detailed info on the 2^nd^ and 3^rd^ pages? 3. Any overall feedback to the design? |
| **Clarity and usefulness of the report:** |
| The first group of questions discuss the clarity and usefulness of the CritCom Report.   1. In the report, we provide an overall score to help you understand how your staff perceive the quality of interdisciplinary communication in your hospital. Do you feel like the score is easy to understand?    1. What does the score mean to you? How do you interpret the score?    2. Based on the report, can you tell what your center's strengths and weaknesses are? 2. Does this report accurately reflect how well communication works in your UNIT?    1. Why or why not? 3. Does it also reflect the quality of communication at your hospital (between units)?    1. Why or why not? 4. How does the written information help you understand how to use the CritCom report? 5. Do you feel you could take action to improve interdisciplinary communication in your unit or hospital based on this report? How? Please give an example based on your report. 6. What other information would you need to help you take action based on this report? |
| **Conclusions** |
| - Is there anything else about taking this assessment and receiving the report you would like us to know or recommend? |
| **Closure** |
| Thank you for participating and for giving us part of your valuable time. We will coordinate with you to offer you information about the analysis of the results of this project.  If you have any comments, questions that were not covered on this session or questions related your center report please email [critcom@stjude.org](mailto:critcom@stjude.org) |

**Table S2: Codebook**

| **Domain** | **Code** | **Definition** |
| --- | --- | --- |
| Interpreting report | Ease of Interpretation | Comments on how easy or hard it is to interpret the CritCom report, including to use it to identify the center's strengths/weaknesses, both for the participant and other members of their team |
|  | Report or Score Interpretation | The participants actual interpretation of their report, including their center's strengths and weaknesses as described by the report (this shows us we need to work on x, or we do a good job with y), anything they were surprised by from their report and if they agree with it. General comments about ease of report interpretation or how one could use the report to understand the center's strengths and weaknesses without specific comments about what those are at their center coded as "ease of interpretation" |
|  | Report Use | Mentions of how the respondents or their team plans to or has used the report to improve communication in their setting; comments about participant's belief or confidence to take action based on the report; include any comments about any actual actions take as a result of this reports |
|  | Additional guidance/information | any comment expressing need for more information from external sources (e.g. St. Jude) about the score/report to successfully implement changes; Additional information that should be provided in the report to improve usability or anything that is missing that should be provided |
| Report Components | Written Material | Comments about the quality of the written text in the report and how it does/does not help with interpretation; includes the "overall communication score" box, "interpreting the results," and "next steps" sections of the report; do not code when discussing written material in other report components; code individual component |
|  | Domain Graph | Comments about the domain averages graphs |
|  | Strengths/Opportunities section | Comments about the strengths/opportunities section of the report; if participants are interpreting their strengths and weaknesses code "interpreting report" |
|  | 2nd and 3rd pages | Comments about the 2nd and 3rd page of the report (excluding "next steps") |
|  | Other individual components | Comments about an individual component of the report not mentioned in the other "report components” codes. General comments about the report should be coded as 'overall report' |
| Overall look and feel | Overall Report | Comments about the overall organization and design of the report, including things that should be adjusted or changed in the report in general, or things that are confusing. Do not code comments about individual components (code one of the 'report components') |
| CritCom | Critcom Comments | Any comments about the CritCom survey, domains, likert scale, or how the survey was administered to staff; does not include any comments about the CritCom report (code other domains) |
| Communication | Challenges of communication | comments regarding what makes communication in or across units challenging; this includes comments about miscommunication, lack of, bad, and/or poor communication; also includes statements about communication as a challenge, statements about disagreements (e.g. about treatment plans or protocols); also includes negative mentions of hierarchy of staff |
|  | Good communication | comments regarding what makes communication in or across units easy or successful; includes statements of positive experiences of communication, successful communication strategies, or overall statements about having good communication between or within units |
|  | Impact on patient care | any comment about how communication impacts care staff members give to patients; this does not include statements on disagreement about treatment plans but instead the actual delivery of care and impact on patient outcomes (both good and bad) (can be double coded with good communication or challenges of communication if segment also mentions specific communication challenges/success/example of specific communication impacting patient care) |
|  | other | any other comment regarding communication between providers, excludes comments only about communication with families |
| Negative | Report Feedback | Double code with any comment of something that is negative or needs improvement in the report or the CritCom tool itself; comments on any part of report that needs changes (double coded) |
